# One hundred years of phase polymorphism research in locusts

**DOI:** 10.1007/s00359-021-01485-3

**Published:** 2021-04-19

**Authors:** Hans-Joachim Pflüger, Peter Bräunig

**Affiliations:** 1 Institut für Biologie, Neurobiologie, Freie Universität, Königin-Luise-Str. 1-3, 14195 Berlin, Germany; 2 Institut Biologie II, RWTH Aachen University, Worringerweg 3, 52074 Aachen, Germany

**Keywords:** Locust polyphenism, Biogenic amines, Motor and sensory behaviour, Chemical ecology, Gene expression

## Abstract

One hundred years ago in 1921, Sir Boris Uvarov recognized that two locust species are one species but appearing in two different phases, a solitarious and a gregarious phase. As locust swarms are still a big problem affecting millions of people, basic research has tried to understand the causes for the transition between phases. This phenomenon of phase polymorphism, now called polyphenism, is a very complex multifactorial process and this short review will draw attention to this important aspect of insect research.

## Uvarov’s discovery of phases

**Fig. 1:**
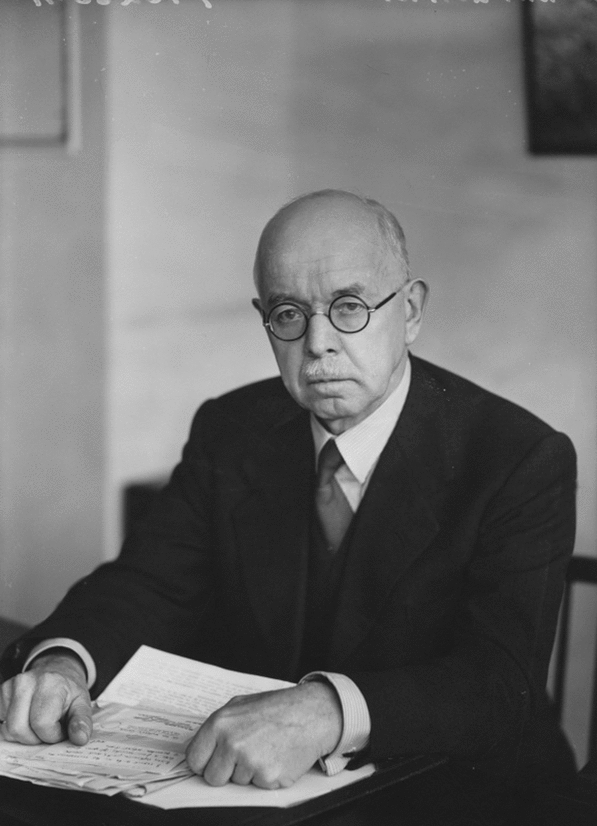
Sir Boris Petrovitch Uvarov, 1886–1970 (by Elliott & Fry bromide print, copyright and courtesy National Portrait Gallery)

Already in the bible, in book Exodus, locusts are reported as one of the plagues of mankind. Who would have guessed that in the twenty-first century, despite of all technical and cultural progress, locusts are still threatening millions of people by destroying their fields and crops, and that swarms may comprise several hundred-millions of individuals and affect areas more than 1000 km^2^. A Russian entomologist, Boris Petrovitch Uvarov (1886–1970), carried out field work from 1911 to 1914 in the Caucasus to solve pending questions surrounding two species *L. migratoria* and *L. danica*. The first world war from 1914 to 1918 prevented Boris Uvarov from publishing his results, and it was only in 1921, one hundred years ago, that he did so in the Bulletin of Entomological Research (Uvarov 1921). In this publication he described and compared the two species *Locusta migratoria* and *Locusta danica* with respect to general and genital morphology, colouration, sexual dimorphism, biology and ecology. In 1912 he and his co-workers collected *L. migratoria* from a big swarm near Sevastopol and studied their offspring in 1913. In his publication (Uvarov 1921) he states: *“it became evident that although the bulk of the larval swarms consisted of migratoria, there were many individuals which were certainly danica, these being different in colouration and showing a tendency to desert the swarms. In the summer of 1913 several specimens of both sexes of very typical danica were isolated in cages, in which copulation and oviposition took place; the eggs hatched without hibernation, as is not uncommon with danica, but so far as we know never occurs in migratoria”.* Uvarov was also able to examine specimens given to him by his friend V. Plotnikov who carried out breeding experiments at the Turkestan Entomological station in Tashkent. Plotnikov’s description of the larvae and adults bred from them was as follows: *“the larvae had in the first stage a dark grey colouration, and not black as in migratoria. In later stages they acquired various colourations—uniformly green, dark grey or brownish—but a number of them had the typical colouring of migratoria, namely, a general reddish-brown colour (sometimes greenish), with velvety black stripes (broad or narrow) along the sides of the pronotal keel and black stripes on the sides of the abdomen. The adults presented no characters typical of danica; the profile of the pronotal keel was usually straight, sometimes even concave. The males were, however, smaller than the females”.* Uvarov (1921), after studying Plotnikov’s specimens, writes:

*“I can only confirm Plotnikov's statement that while the parents are all very typical danica, save that not all of them have the hind tibiae red (which character is not quite constant in that form), their direct offspring are on the contrary all well-defined migratoria, though a few of them have the tibiae red, as is sometimes the case in this form”.* More of such experiments and some detailed geographical considerations brought Uvarov to formulate his theory of phases. He states: *“that the three forms cannot be separated specifically and that they represent taxonomic units of lower grade than the species, which must be called, according to the law of priority, L. migratoria L. They are, however, quite distinct from each other, though connected by transitional forms”.* He argues against using the term “morpha” (morph) and suggests “phase” instead. Well ahead of his time he also suggests for the control of locust outbreaks that *“the theory of phases suggests the theoretical possibility of the control of migratoria by some measures directed not against the insect itself, but against certain natural conditions existing in breeding regions which are the direct cause of the development of the swarming phase.”* Therefore, it is due to the studies of Boris Uvarov (1921), and his friend V. Plotnikov, that the appearance of locusts in two phases, the solitarious and the gregarious one, was recognized for the first time. Later in England, Sir Boris Uvarov (Fig. 1) continued his studies on locusts (Uvarov 1977) as the director of the Anti-Locust Research Centre in London from 1945 to 1964.

## Biology of phases: differences in behaviour

**Fig. 2:**
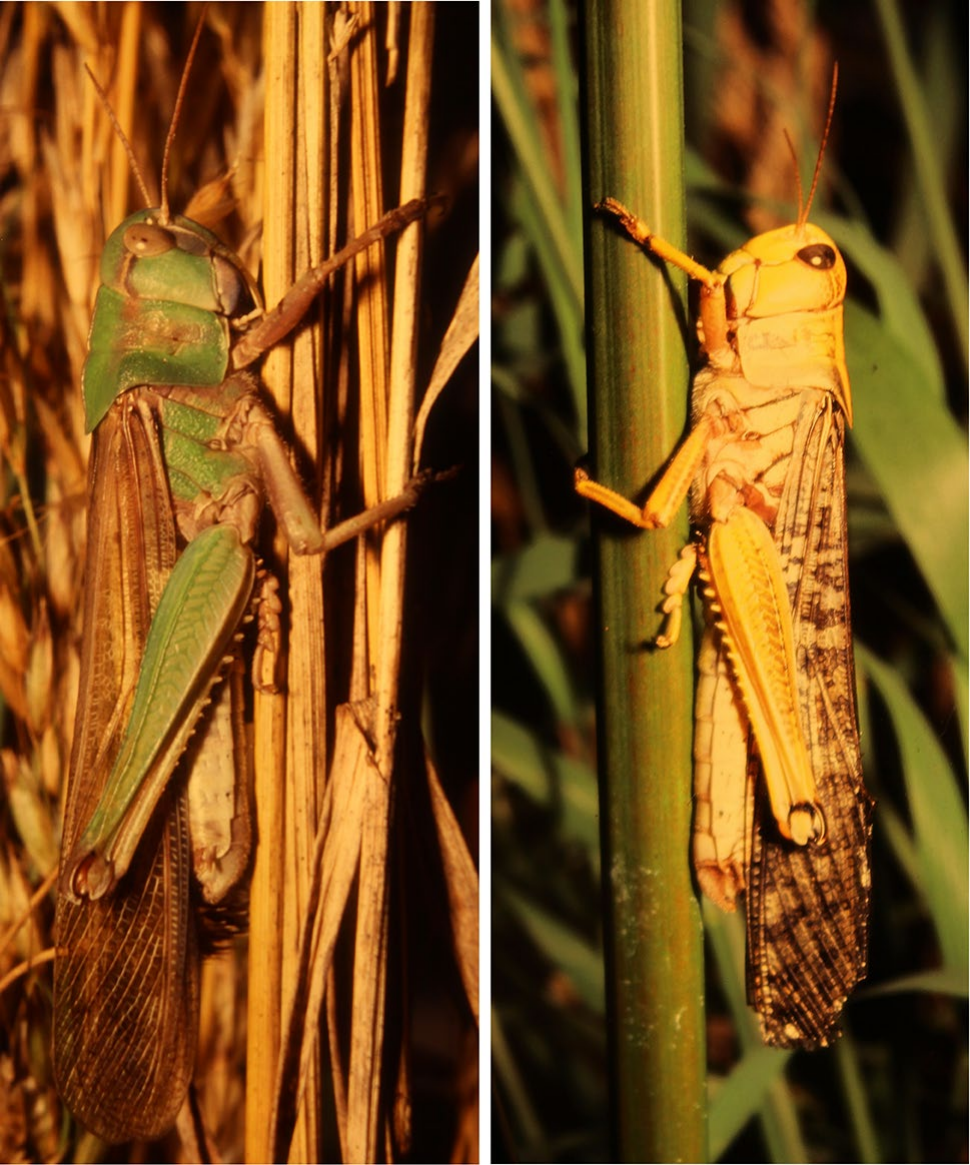
Two males of *Locusta migratoria* from a breeding experiment in the lab of Peter Bräunig. The one on the left is in the solitarious, the one on the right in the gregarious phase. Please note differences in colour, shape of pronotum, and length of hind leg femur. (Photographs: Willi Maile)

The reason, why *L. migratoria* and *L. danica* may have been considered two separate species were differences with respect to colouration of adults and nymphs (Fig. 2), behavioural differences with *L. danica* nymphs avoiding gregarization and *L. migratoria* nymphs joining other hopper bands, and differing preferences of breeding places. However, male genitalia, a morphological feature employed for exact species determination in insects, were of very similar shape, and artificial breeding experiments yielded nymphs with features of *L. danica* that had hatched from *L. migratoria* egg pods (Uvarov 1921). Nowadays it is clear that approx. 20 species of grasshoppers world-wide, taxonomically not closely related in most cases, may have the potency to form swarms and exhibit different phases (a phenomenon now called polyphenism, meaning the same genotype exhibiting several phenotypes; Simpson and Sword 2008; Pener and Simpson 2009). The species studied most are the migratory locust, *Locusta migratoria*, and the desert locust, *Schistocerca gregaria*. Detailed studies in both species, revealed that all kinds of intermediates between the phases can be found and that transitions between phases can occur at any stage and in both directions, all depending on the density of populations (Pener 1991; Pener and Simpson 2009). Each of the 20 species must be regarded separately with respect to phase changes as clear species-specific differences and even geographical variants within one species may exist (Pener and Simpson 2009). For example, solitarious individuals of *Schistocerca gregaria* possess six larval instars (stages as nymphs) whereas gregarious ones only have five larval instars. This is different in the migratory locust, *Locusta migratoria*, where both phases seem to have the same number of instars. In the desert locust (*Schistocerca gregaria*), very obvious differences between the phases exist. Solitarious desert locusts are shy, are more sedentary and do not move much, and with their green or brownish cryptic colour hide during the day. They also avoid other locusts, except for mating of course, and if they migrate, they are reported to fly at night (Uvarov 1977). In contrast, gregarious desert locusts are not cryptic at all but reveal anti-predator warning colours in bright yellow and black. Gregarious animals are very active, and they aggregate both as nymphs (“marching hopper bands”) and adults (groups, swarms), and they fly by day and roost over-night in trees. Differences are also observed between grooming frequency, resting time and the time spent near a group of other locusts (Rogers et al. 2014). In addition, walking speed differs as well as the walking posture: solitarious walk slowly with body held low to ground whereas gregarious walk rapidly with body held high over ground and hind legs also exhibit different trajectories during walking. A most interesting difference was described by Simoes et al. (2013) with respect to olfactory learning: 70% of all tested locusts preferred vanilla odour to lemon. If vanilla was now paired with an aversive stimulus, only 34% of solitarious locusts still preferred vanilla in contrast to the majority of gregarious locusts, 59%, that still preferred vanilla. Even more astonishing is that solitarious locusts avoid feeding on Black Henbane (*Hyoscyamus niger*) which contains the alkaloid hyoscyamine whereas gregarious locusts feed on this plant. Hyoscyamine, therefore, acts either aversive or appetitive depending on phases (Despland and Simpson 2005a, b). If food containing hyoscyamine is paired with an odour in an olfactory learning experiment, solitarious locusts develop a strong aversive memory for the paired odour in contrast to gregarious locusts (Simoes et al. 2013). Therefore, an aversive memory for an odour is eased when a locust becomes gregarious. It is now clear that polyphenism is a multifactorial phenomenon which is not uni-causal but involves changes in morphology, anatomy, colouration, development, reproduction, physiology, biochemistry, molecular biology, behaviour as well as all aspects of ecology including chemical ecology (Hassanali et al. 2005; de Loof et al. 2006; Despland and Simpson 2006; Simpson and Sword 2008; Pener and Simpson 2009; Cullen et al. 2017), and all these changes may have different time scales with those of behaviour being the fastest to occur (Ayali 2019).

## Biology of phases: sensory stimulation

A very good test for phase was designed by Simpson et al. (1999) and later systematically applied by Anstey et al. (2009) in which the locusts entered an arena which had a group of other locusts displayed on one side and on the other side an empty space. The choice of the individual locust in its approach to either side ultimately provided a “phase score” of solitariousness, or gregariousness respectively. From previous observations it was clear that one of the major stimuli in the transition of phases were tactile and olfactory stimuli. Indeed, crowding a solitarious individual with other nymphs, or regularly touching the hind femur of such a nymph, or exposing the nymph to the smell and sight of other nymphs caused the tested individuals to turn from solitarious to gregarious (Rogers et al. 2003; Anstey et al. 2009).

## Biology of phases: the role of biogenic amines

Tactile stimuli were shown to increase serotonin concentrations in thoracic ganglia (Burrows et al. 2011), and either injecting serotonin or precursors, or receptor agonists into solitarious locusts induced gregarious behaviour. Correspondingly, injection of serotonin receptor antagonists or synthesis inhibitors prevented solitarious locusts to become gregarious (Anstey et al. 2009). Other biogenic amines are affected as well: Isolating *Schistocerca gregaria* increased dopamine levels in the brain (Alessi et al. 2014). Previously, Ma et al. (2011) found in a genome-wide gene expression profiling in solitarious and gregarious nymphs of *Locusta migratoria* that catecholamine pathways, particularly those of dopamine, are upregulated in the gregarious phase. For *Locusta migratoria,* Guo et al. (2015) report that dopamine signalling via Dop1-receptors seems to play a role in gregarization whereas signalling via Dop2R mediates the opposite, solitarious behaviour. With respect to tyramine and octopamine, tyramine titres decrease when *Schistocerca* gregarious nymphs are isolated (Rogers et al. 2014) and octopamine titres decrease in the metathoracic ganglion when adult gregarious *Schistocerca* are isolated (Alessi et al. 2014). When body volatiles of *Locusta migratoria* are tested (Ma et al. 2015), solitarious locusts when crowded are attracted and gregarious locusts when isolated are repelled. Both, tyramine and octopamine signalling were correlated with this behavioural switch. Using RNAi-mediated knockdown of receptors, Ma et al. (2015) showed that OARa and TAR are involved. Activation of OARa signalling in solitarious locusts caused a behavioural shift from repulsion to attraction. Enhancement of TAR signalling in gregarious locusts resulted in a behavioural shift from attraction to repulsion. Interesting differences were also found with respect to brain size: gregarious *Schistocerca* have a 30% larger brain size, for example larger optic lobes, despite their smaller body size compared to solitarious individuals (Ott and Rogers 2010). In solitarious *Schistocerca* the antennal lobes are larger as they possess more olfactory receptor neurons than gregarious ones (Anton and Rössler 2020).

## Biology of phases: pheromones

An impressive number of publications deals with the search for pheromones, in particular aggregation pheromones (see reviews of Pener and Yerushalmi 1998; Ferenz and Seidelmann 2003; Hassanali et al. 2005; Pener and Simpson 2009; Cullen et al. 2017). Others deal with differences between the phases in their chemosensory equipment and olfactory processing of pheromonal signals (Ochieng et al. 1998; Anton et al. 2002). It is now clear that chemical ecology plays an important part in the life of locusts and that a bouquet of various odorants may act at different times during their development and adult maturation and, therefore, may have different effects at different times. Substances were isolated from the body of locusts, from faeces, from egg pods or from the soil in which mass oviposition took place, and then tested by gas chromatography together with mass spectrometry or in some cases also with electroantennograms. Thus, locusts possess a bouquet from odorants such as hexanal, octanal, nonanal, decanal and the corresponding acids including valeric acid (pentanoic acid) and faecal compounds are guaiacol, phenol and indole. Most interesting are volatiles of adult solitarious or gregarious *Schistocerca* males. In both phases, anisole, benzaldehyde, guaiacol and phenol were identified and vetratrole was present in trace amounts in solitarious males (Njagi et al. 1996). The major compound of gregarious *Schistocerca* males and absent from solitarious males was phenylacetonitrile (PAN) or benzyl cyanide. Detailed analyses by Seidelmann et al. (2000; 2003) and Seidelmann and Ferenz (2002) showed that this substance is released from wings and legs and can be considered a mating regulator or “courtship inhibiting pheromone” as it prevents additional mating between females and other males. In a different species *Schistocerca piceifrons*, Stahr and Seidelmann (2016) showed that females preferentially mate with males emitting a high concentration of the volatiles phenylethyl alcohol (PEA) and (*Z*)-3-nonen-1-ol (3-NOL) and this also affected successful hatching of their larvae. Recently, a careful study of Guo et al. (2020) identified 4-vinylanisole or 4-methoxystyrene as an aggregation pheromone in *Locusta migratoria*. This substance is both attractive for nymphs and adults and seems to act via particular odour receptors (OR35). From the above it is clear that locusts, solitarious and gregarious, experience a wealth of chemical cues which are used for communication much more complex than previously anticipated.

## Biology of phases: genomic approaches

The final and latest studies on locusts are concerned with genes and their differential expression in the different phases. Such studies suffer from the fact that the genome of *Schistocerca gregaria* so far is the largest insect genome sequenced and assembled to date. In total, 18,815 protein-encoding genes are predicted in the desert locust genome, of which 13,646 (72.53%) obtained at least one functional assignment based on similarity to known proteins (Verlinden et al. 2020). In *Locusta migratoria*, a similar large number of genes, 17, 307, was predicted (Wang and Kang 2014; Wang et al. 2014). In a study of the transcriptomes of solitarious and gregarious *Locusta migratoria,* 214 transcripts exhibited differences (more stress response related genes in gregarious adults, more oxidative stress resistance genes in solitarious adults; Badisco et al. 2011). Upregulated genes in gregarious locusts were heat shock proteins, proteins which give protection from infections, greater abundance of transcripts for proteins involved in sensory processing, nervous system development and plasticity, and in general, genes that play a role in stress responses. Upregulated in solitarious locusts were genes related to anti-oxidant systems, detoxification, anabolic renewal, and in general, protection against slowly accumulating effects of ageing. Such genomic studies could be very interesting in the genus *Schistocerca* as many species live in North- and South America and only a few of these show polyphenism and the potency to form swarms. The different lifestyles of these closely related species may offer a very good opportunity to identify more genetic or epigenetic factors relevant for polyphenism (Ernst et al. 2015).

## Concluding remarks

Research of Boris Uvarov in Russia published 100 years ago still impacts on today’s entomologists, neurobiologists, and ecologists, because locust swarms still threaten the nutritional basis of millions of people in many parts of the world. As recent outbreaks in Eastern Africa indicate, these problems might increase in the future due to climate changes affecting ecosystems and changes due to agricultural land use. Unfortunately, the affected areas belong to the most fragile and vulnerable landscapes. In addition, civil wars, ethnic unrest and often a lack of overseeing government add to the still pending problems with locusts (Meynard et al. 2020). It is now clear that phase polyphenism can have many causes and is a multifactorial process. Based on the pioneering work of Boris Uvarov we have acquired a much better knowledge of phase polyphenism but our understanding is far from complete. Definitely, this merits further research, particularly with respect to now available methods that allow addressing influences of epigenetic modifications, changes in the microbiome, and for performing modelling studies at the level of populations (Wang and Kang 2014; Ernst et al. 2015; Ayali 2019).
